# Enhanced Oil Spill Remediation by Adsorption with Interlinked Multilayered Graphene

**DOI:** 10.3390/ma12142231

**Published:** 2019-07-10

**Authors:** Marco Vocciante, Antonio Finocchi, Alessandra De Folly D′Auris, Alessandro Conte, Jacopo Tonziello, Annalisa Pola, Andrea Pietro Reverberi

**Affiliations:** 1 DCCI, Department of Chemistry and Industrial Chemistry, Università degli Studi di Genova, Via Dodecaneso 31, 16146 Genova, Italy; 2 Eni S.P.A., Upstream and Technical Services, Via Maritano 26, 20097 S. Donato Milanese (MI), Italy; 3 Directa Plus S.P.A, Via Cavour 2, 22074 Lomazzo (Co), Italy

**Keywords:** water remediation, hydrocarbons removal, selective adsorption, innovative adsorbents, super-expanded graphite

## Abstract

The performances of an innovative material based on graphene multilayers in a 3D structure similar to expanded graphite, Grafysorber^®^ G+ (Directa Plus), have been tested via in field applications on a real contaminated site. Several experimental tests were performed using Grafysorber^®^ inside adsorbent devices (booms and pillows) to treat waters polluted by oil. The experimental campaign was carried out with the aim of comparing the performances of Grafysorber^®^ with those of polypropylene (PP), which is the material used worldwide in case of water oil spill clean-up activities. The results achieved have confirmed a considerably higher selective adsorption capacity of Grafysorber^®^ compared to PP, and configure the new material as a promising alternative to standard materials in enhancing oil spill remediation by selective adsorption.

## 1. Introduction

Any accidental or deliberate release of liquid hydrocarbons into the environment is termed as an oil spill. The problems arising from hydrocarbon releases in port areas and in the open sea are very different. In the latter case, numerous oil spills have shown the extent of the damage inflicted on the environment when a large volume is released over a short period of time [1]. These events are quite difficult to contain and are responsible for catastrophic effects on the environment. It was estimated that over 100 million gallons of crude oil spill into seawater each year [2], with a huge negative impact not only on aquatic species, but also on other animals and vegetation along the coasts. Large and catastrophic spills belong to the category of high severity and low probability events and pose a serious ecological risk possibly resulting in long-term environmental impacts depending upon the quality, chemistry and properties of oil and its sensitivity to biological resources impacted [3]. However, minor spills connected to failures of pipelines connecting port storage areas and process plants can still give rise to severe accident scenarios and environmental degradation linked to the displacement of contaminants in the soil [4,5], with transboundary effects, in the case of watercourses, or international seas, as recently demonstrated by Vairo et al. [6].

The number of large oil spills has declined over the years, possibly due to an increased awareness in society coupled with stringent policy enforcement by governments [7]. Nonetheless, devastating oil spills are still occurring, such as the Exxon Valdez accident in 1989 [8], the Deepwater Horizon spill in 2010 [9] or the MT Dawn Kanchipuram in 2017 [10], to name a few.

Mitigation associated with oil spill impacts depends largely on the implementation of effective contingency plans, which should incorporate source identification, simulation of the respective dispersion patterns, and characterization of affected areas, where the cleaning operations are mandatory [11].

Several approaches were developed aimed at the different phases of a typical cleaning operation and can be classified according to the nature of the intervention in chemical (gelling agents, dispersive materials), biological, physical (skimmers, barriers and sorbents) and thermal (burning) methods. Based on their modes of action, physical countermeasures can be defined as active or passive [12], yet sorbents are hybrid systems, as they can be used for active removal as well as containment, and are particularly effective in recovering traces of oil from both land and water [13].

To date, the remediation of aqueous solutions often relied upon adsorption as a simple, easy to implement and particularly effective method for the removal of a wide variety of contaminant, both organic [14] and inorganic [15]. This is even more interesting when it is possible to exploit low-cost adsorbent materials from waste [16] with a dual benefit for the environment, in line with operational guidelines such as the Circular Economy and the “near-zero discharge” of hazardous waste (see, for example, [17]) established by the latest European laws.

In the field of research aimed at the management of oil spills, absorbent materials have recently attracted considerable attention due to their potential capacity to recover oil, lack of secondary pollution, in case of complete collection after use, possible scalability [18] and simple application. A wide variety of materials can be used, including organic materials, such as bark, peat, sawdust, straw and wool, inorganic compounds such as vermiculite, pumice and alumina [19], and synthetic materials such as polypropylene (PP) and other polymers.

An ideal oil sorbent should fulfill several requirements, including sufficient hydrophobicity, high absorption and retention capacities, good selectivity, high oil absorption rate, good mechanical resistance, reusability, buoyancy, low cost, abundance and biodegradability [20].

Nowadays, oil sorbents with various characteristics are produced by manipulating natural and synthetic materials. Synthetic materials are generally more efficient in recovering oil, and their better practical and mechanical characteristics as well as the possibility of reusability can be tuned and artificially optimized [21]. On the other hand, natural sorbents have a low environmental impact due to their biodegradability, are generally abundant in nature and consequently have a lower cost [22].

These materials can be assembled in various forms depending on their composition and use. They are generally found as loose materials that are enclosed in cushions or booms, absorbent sheets or loose fibers intertwined with each other.

As mentioned, synthetic materials are those that have the best performance; among these, to date polypropylene (PP) is the most used and it is precisely this material that has been taken as a comparison in the tests described below. Unfortunately, PP usage has several disadvantages, such as lack of biodegradability, difficult recovery of the adsorbed oil—due to water retention—and expensive landfill disposal of the used material [23]. Furthermore, it is inefficient in case of reclamation of hydrocarbons present at low concentrations or as an iridescent monolayer on the aqueous surface. In this context, developing a technique for the production of natural-based oil sorbents with sorption properties comparable to those of synthetic ones and, at the same time, preserving their own advantages (for example, the biodegradability), would be of great interest as well as an obvious starting point for improving the sustainability of the intervention.

Grafysorber^®^ G+ (Directa Plus, Como, Italy) is a material based on expanded graphite, a substrate having excellent properties as a sorbent for saturated and unsaturated hydrocarbons, both in liquid and in gaseous form, with promising applications in safety science [24] and in landfill management technology [25]. The expanded form of graphite, derived from the expansion of graphite flakes, has aroused great interest because of its high capacity of adsorption assisted by extremely fast kinetics, the absence of toxicity and non-flammability. The usefulness of expanded graphite (EG) for “oil on water” or “pure oil” sorption was first discovered by the research group of Inagaki and Toyoda [26,27,28,29,30]. In their research, they reported the maximum adsorption capacity for different oils (up to more than 80 times the weight of the EG in case of A-grade heavy oil) [26], and deeply investigated several aspects such as effects of bulk density of EG on the sorption behavior [27], recovery of heavy oil and recycling of EG [28], trials for practical applications [29], etc. In this regard, its buoyancy and the possibility of recovering the adsorbed oil [30] represent further undeniable advantages, as well as the substantial independence of adsorption from salinity content [31], which confirms that EG is suitable for oil leakage accidents under seawater conditions [32].

Furthermore, the addition of additives and reaction aids is not necessary [33,34], and the adsorption capacity of expanded graphite (EG) increases with increasing expansion volume [35], thus suggesting the possibility of even greater performance.

However, it should be noted that most of these studies are carried out on a laboratory scale and treat the adsorption of only one type of oil at a time.

On the contrary, while laboratory studies on Grafysorber^®^ G+ are still in progress at our group, in the present work the performances of the new material are directly tested in the field, within adsorbent devices (booms and pillows). This means on the one hand having to deal with a complex contamination from hydrocarbons, on the other hand to operate on much larger scales, with problems of confinement of the material very different from what is observed on a laboratory scale.

The reasons for this choice lie in the opportunity of improving the remediation activity of a site of interest polluted by a strong presence of hydrocarbons where PP sorbents are currently adopted, thus also allowing the possibility of a direct comparison between the real performances of the two materials.

## 2. Materials and Methods

### 2.1. Site Description

The site of interest, located in Nigeria, has registered a significant increase of oil spill events due to sabotages and thefts on the pipelines used to collect and transport the oil.

PP pillows and booms are currently used to sorb and contain spills in a land area and in a channel flowing to Atlantic Ocean.

### 2.2. Adsorbent Materials

**Figure 1:**
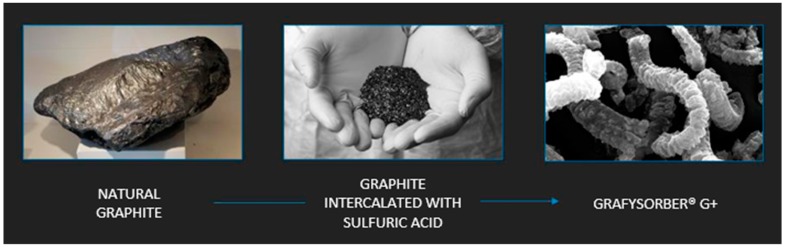
Production process of Grafysorber^®^ G+.

Grafysorber^®^ G+ is a material based on graphene multilayers forming a 3D structure similar to expanded graphite, a technological product patented by Directa Plus (Como, Italy) [36] having an apparent density between 2.3 and 3.5 g/L. The chemical intercalation of natural graphite with sulfuric acid, followed by thermal expansion in an inert gas plasma, is the basis of the production process designed to push the expansion of graphite to its extreme limits (Figure 1). This concept is practically quantifiable in an expansion ratio of about 300 units, with respect to the average values of the order of 100 obtainable through standard methods [37,38].

A subsequent exfoliation phase leads to the separation of graphene packets in a structure similar to an accordion (60–300 µm in diameter and length up to 5–6 mm), with few layers of graphene weakly bound along the edges, resulting in low apparent density, leading to a considerable adsorption capacity for oils and hydrocarbons. The oil adsorption capacity of the loose material, evaluated by laboratory tests according to the American Society for Testing and Materials (ASTM) 726-12 method (which depends on oil viscosity), is around 94 g/g, reaching a maximum of 122.8 g/g for a synthetic engine oil (I-sint 10W40) [39].

Grafysorber^®^ G+ is chemically and biologically inert; in addition, the highly crystalline nature guarantees the thermal stability of the material up to 600 °C in air and provides a highly hydrophobic behavior.

Grafysorber^®^ G+ is a coarse powder with a three-dimensional, accordion-like, quaternary structure. Due to its buoyancy, it can be used in loose form if the application allows it (e.g., in the treatment of industrial or refinery wastewater consisting of a two-phase mixture that can be easily confined inside tanks); once saturated, it can be easily handled and/or removed.

However, owing to its light weight, it is used with difficulty in the event of oil spills in open environments such as the sea, lakes and rivers. Under these conditions, it is necessary to confine the material inside a non-woven fabric (NWF) made of polypropylene, which allows the oil to penetrate inside it. A preliminarily assessment of the properties of the NWF used did not show any evidence that the diesel is retained by the NWF.

To compare the oil sorption efficiency, samples of PP were purchased from a European company that produces oil sorbent devices. Two different types of confining elements, respectively a fabric and a needle felt, were tested to determine the impact of the media structure to the efficiency of the boom, both in terms of sorbent material confinement and oil affinity.

### 2.3. Oil Spill Composition

**Figure 2:**
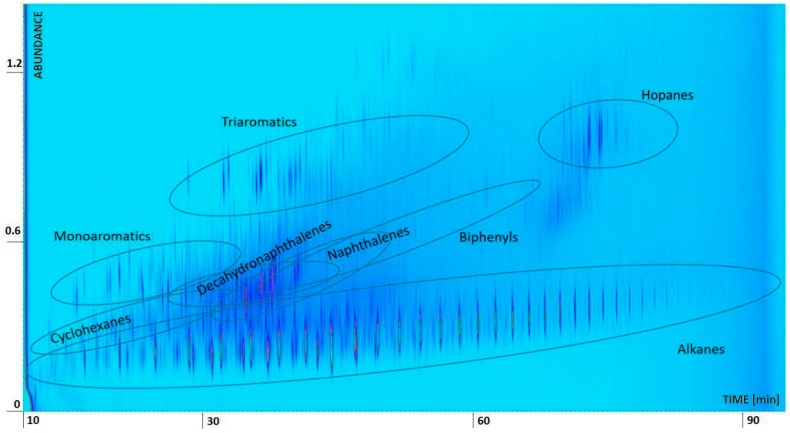
Comprehensive Two-dimensional Gas Chromatography (GC × GC) analysis of the oil.

**Figure 3:**
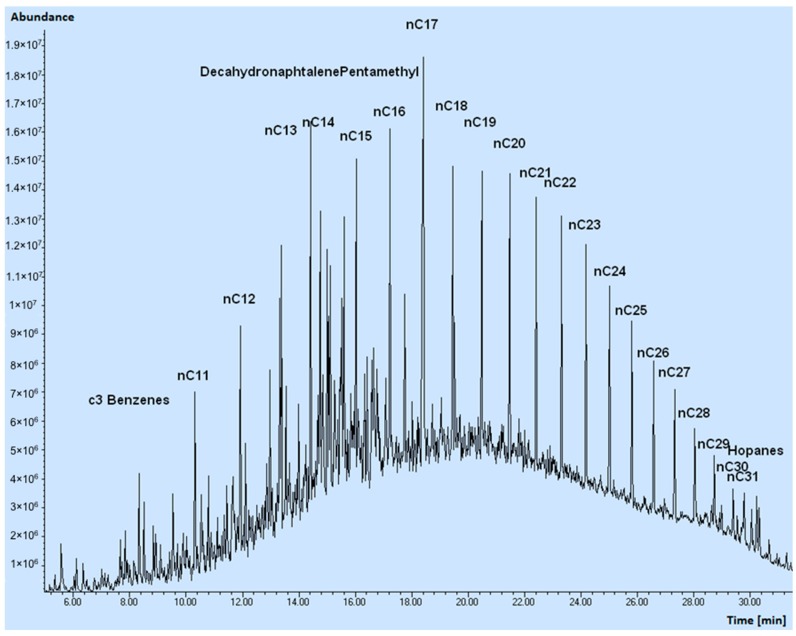
Total ion current chromatogram (GC-MS analysis) of the oil.

The oil was characterized in physical terms by determining the density and viscosity values (Table 1), and chemically through Comprehensive Two-dimensional Gas Chromatography GC × GC (Figure 2) and Gas Chromatography Mass Spectrometry GC-MS analyses (Figure 3).

The density and viscosity values were determined by using a Stabinger meter and measuring at 45, 25 and 20 °C. It was not possible to measure the density at 15.5 °C, as required for the calculation of the American Petroleum Institute (API) grade, since at 20 °C the measurement began to be unstable, thus suggesting that a phase change occurs around this temperature.

For the GC analyses, samples were diluted in dichloromethane (1:1000). The GC × GC investigation (Agilent Technologies 7890B, Santa Clara, CA, USA) was performed by using a split (1/10) injection mode, a Flame Ionization Detector (FID) detector, and two different columns, namely a J&W CP9092 (Agilent Technologies, Santa Clara, CA, USA) (length 30 m, diameter 0.25 mm, film 0.1 µm) and a Zebron 7HG-G025-11 (Phenomenex, Torrance, CA, USA) (length 5 m, diameter 0.25 mm, film 0.25 µm). The heating ramp used ranged from 50 to 400 °C with a speed of 4 °C min^−1^. GC × GC identifications were made based on data acquired during a previous analysis of different groups of gas oils and crude oils, as well as by referring to the GC-MS analyses carried out on the same oil.

The GC-MS analysis (Agilent Technologies 6890, Santa Clara, CA, USA) was operated in splitless injection mode, using ionization by electronic impact at 70 eV. Identifications were realized, when possible, by investigating the MS spectrum obtained. The complexity of the samples, together with the co-elution of different products, makes it difficult to get a reliable recognition for most minor products. The quality of the spectrum was improved either by subtracting the fund or attributing the general name of «Cn derivatives» (indicating with Cn the presence of one or more methyl or alkyl groups, for a total sum of ‘n’ carbon atoms) to a group of peaks and taking as confirmation of the attribution the similarity of the spectrum and the belonging to a group recognizable in the reconstructed chromatogram. A more precise attribution of individual isomers was beyond the scope of the study.

The estimated majority classes are summarized as follows: alkanes, cyclohexanes, mono-aromatics, naphthalenes, biphenyls, tri-aromatics, decahydronaphthalenes and hopanes. As shown in Figure 2, GC × GC signals appear as elongated spots; the concentration, proportional to the peaks volume (area per height), is represented by the variation in color and increases from blue to yellow.

As anticipated, a rather complex GC-MS chromatogram was obtained, characterized by the main peaks of the normal-alkane series, by a series of peaks identified as cyclohexanes, mono-aromatic agents (ethylbenzene, xylenes, C3-C5 benzenes), di-aromatics (naphthalene and C1-C4 naphthalenes), bi-phenyls, tri-aromatics (phenanthrenes and anthracenes), decahydronaphthalenes and hopanes. Below these peaks, a continuous noise due to non-separate products is evident (the GC × GC approach allows a better separation).

### 2.4. Experimental Campaign

**Figure 4:**
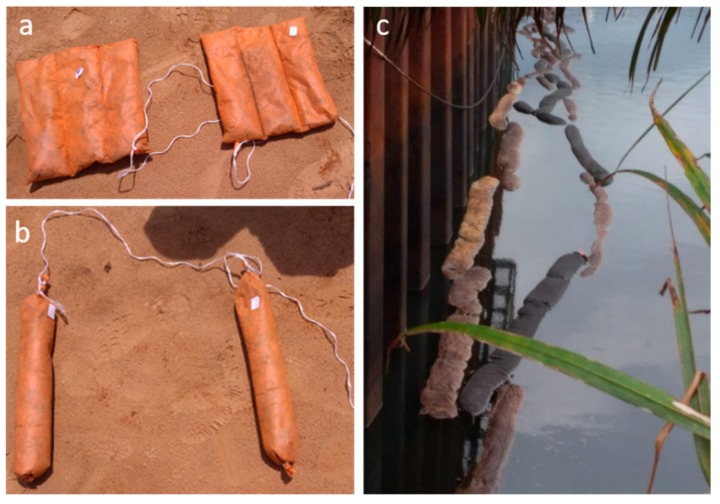
(**a**) Grafysorber^®^ pillows and (**b**) Grafysorber^®^ booms for American Petroleum Institute (API) oil-water separator test; (**c**) chains of Grafysorber^®^ and polypropylene (PP) booms in the channel.

Sorbent materials were deployed in the polluted channel by covering its entire width with chains composed of Grafysorber^®^ and PP booms linked to each other alternately. As a second test, two pillows and two booms, made with Grafysorber^®^ and PP (Figure 4), were deployed for 21.5 h in an API oil-water separator installed upstream of the channel, in order to study the sorbents behavior in a system completely filled with oil and thus under conditions of fast adsorption kinetics. At the end of the test, pillows and booms were removed from the API oil-water separator, weighed after dripping and sectioned to withdraw the material inside. A sample of oil and eight samples of materials (4 of Grafysorber^®^ and 4 of PP) were respectively collected from the separator and from sectioned sorbents to be analyzed. Oil analysis was discussed in the previous section. Since the test was carried out in the separator, where only the oil phase is present, pillows and booms adsorption capacities were measured by simply comparing their final and initial weights. Obtained results are shown in Table 2.

## 3. Results and Discussion

Adsorption tests data related to the polluted channel are not yet available, therefore only those related to the experimentation in the API oil-water separator are discussed in this work.

### 3.1. Thermogravimetric Analysis of Samples

The amount of oil adsorbed per unit weight of adsorbent can be measured through a thermogravimetric analysis (TGA). During the TGA tests (TGA/DSC1, Mettler-Toledo International, Greifensee, Switzerland), it is assumed that the adsorbed components evaporate under heating in an inert environment (nitrogen), without leaving appreciable residues. Moreover, the possible pyrolysis of products is minimized by performing a slow heating (10 °C min^−1^) and reaching a relatively low final temperature (550 °C). In addition, a high flow of nitrogen (50 mL/min) is used to remove the hydrocarbon vapors formed thereby (before they have time to burn), thus protecting the sample from the formation of non-volatile pyrolysis products. The reliability of the method was investigated during previous tests carried out with known amounts of Grafysorber^®^ saturated with different amounts of diesel fuel; at the end of each TGA run, the residual sample weight matched the initial Grafysorber^®^ weight. Since all assumptions were widely confirmed by the results, estimates of the adsorbed-to-adsorbent ratio were obtained by simply evaluating the adsorbent mass as the residue of the TGA and the adsorbed amount as the mass loss during the measurement.

Regarding TGA analyses carried out on samples coming from the Nigerian field, the unknown content of non-volatile compounds in the oil might have led to an overestimate of the amount of adsorbent. However, even if this overestimated quantity was present, the evaluation of the adsorbed-to-adsorbent ratio should not be affected significantly, as both Grafysorber^®^ and PP adsorbed the same oil.

### 3.2. TGA Tests on Grafysorber^®^

**Figure 5:**
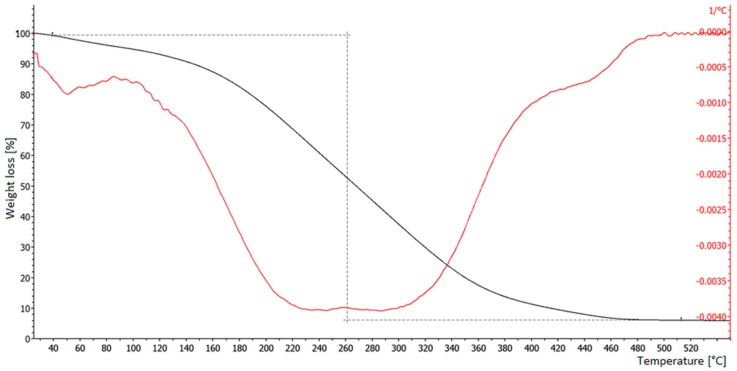
TG analysis of a Grafysorber^®^ sample. Black line: thermogravimetric analysis (TGA) (weight loss); red line: first derivative of the TGA.

As shown in Figure 5, the TGA curve of a Grafysorber^®^ sample is fairly smooth, while the first derivative of the curve shows deviations that likely correspond to the evaporation of products at different temperature.

As shown in Table 3, TGA measurement replicates were carried out on one and the same Grafysorber^®^ sample named “Boom-1”, obtaining an adsorbed-to-adsorbent ratio ranging from a minimum of 11.82 mg mg^−1^ to a maximum of 22.43 mg mg^−1^.

### 3.3. TGA Tests on Polypropylene

**Figure 6:**
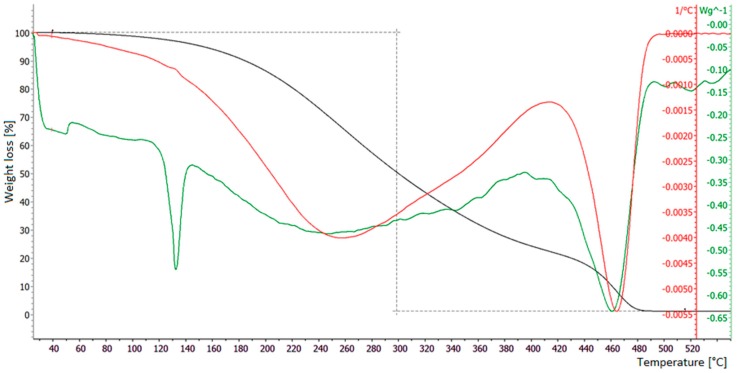
TG analysis of a PP sample. Black line: TGA (weight loss); red line: first derivative of the TGA; green line: DSC (differential scanning calorimetry).

The same analyses were performed on PP samples. Differences exist between TGA curves for Grafysorber^®^ and PP, due to their different physical properties. In fact, PP melts at a temperature around 130 °C and evaporates almost completely leaving a waxy residue on the bottom of the measuring crucible at the end of the TGA run (Figure 6). This residue presents a reddish brown color, thus suggesting that part of the oil might have been incorporated in the waxy component.

**Figure 7:**
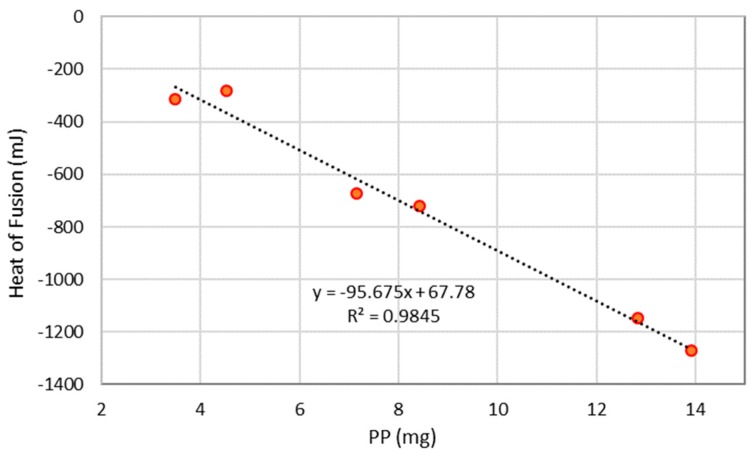
Calibration curve relating the PP heat of fusion to the amount of pure PP.

It was not possible to determine a univocal relationship between the quantity of PP and the weight of remaining residue. However, the TGA differential scanning calorimetry (DSC) curve presents a well-defined and repeatable heat absorption peak, centered at a temperature of 130 °C, related to the PP heat of fusion. Assuming, as a first approximation, that the total heat absorbed by the PP during the fusion is not affected by the presence of hydrocarbons, a calibration curve (Figure 7) using the heat of fusion of pure PP samples was prepared by accurately weighing different amounts of PP and carrying out the TGA/DSC investigation (Table 4).

Then, TGA/DSC analyses were performed with six replicates on a PP sample coming from Nigeria (named “Boom_5”) with the aim of determining the heat of fusion that is related to the amount of PP according to the calibration curve (Figure 7).

From a comparison of data in Table 3 and Table 5, it is clear that under the investigated experimental conditions, PP adsorbed between 4 and 6 times its own weight, while Grafysorber^®^, in the same conditions, absorbed a quantity of oil between 12 and 22 times its own weight. Taking into account that the TGA was performed on small amounts of samples and considering the issue of the melting of PP, obtained results are in reasonably good agreement with those obtained by direct weighing (Table 2).

## 4. Conclusions

Grafysorber^®^ is an innovative oleophilic/hydrophobic material with high adsorption capacity.

The proprietary technology G+ (Directa Plus) gives unique characteristics to the material, which is not simply expanded graphite, but a three-dimensional structure consisting of layers of graphene weakly bound assuming an accordion-like shape, leading to top-notch adsorbent capacities towards the oil phase.

In the present work the performances of the new material are directly tested in the field, within adsorbent devices (booms and pillows), for reasons of opportunity.

The need for operating on much larger scales and dealing with a complex contamination from hydrocarbons allowed a direct comparison between the real in situ performances of the G+ and the PP sorbents.

Preliminary results obtained by testing Grafysorber^®^ under the conditions described above confirm that it can be used to treat water presenting an oily separated phase, allowing oil to be easily removed with better efficiency compared to the PP. The greater adsorption capacity turns into smaller quantities of adsorbent to be used and therefore to be disposed of, while the greater selectivity makes it possible to recover a large part of the adsorbed oily phase. All this without considering the possibility of further use of the regenerated material, although with reduced performance.

At the same time, the adsorption capacity of G+ in the test conditions, while remaining in every condition superior to those observed with the PP, appears to be considerably inferior to that achieved in laboratory studies on our and other EG materials reported in the literature. Further laboratory investigations are in progress to deepen this and other aspects and improve efficiency in the field.

In conclusion, though further improvements seem possible, the results achieved so far in treating a real site polluted with hydrocarbons by ensuring a greater effectiveness of the oil spill clean-up process makes Grafysorber^®^ a remarkable step forward towards a sustainable oil spill remediation.

## Figures and Tables

**Table 1:** Oil characterization.

Temperature (°C)	Density (g cm^−3^)	Dynamic Viscosity (mPa·s)	Cinematic Viscosity (mm^2^ s^−1^)
45	0.8795	9.42	10.71
25	0.8929	21.14	23.67
20	0.8969	72.14	80.43

**Table 2:** Samples for tank tests.

Adsorbent	Format	Size (cm)	Initial Weight (g)	Final Weight (g)	Adsorption (g/g)
Grafysorber^®^	Boom	50 × 7.5	66	1500	22.73
		50 × 7.5	67	1500	22.39
	Pillow	50 × 50	106	2200	20.75
		50 × 50	102	1800	17.65
Polypropylene	Boom	50 × 7.5	185	1600	8.65
		50 × 7.5	184	1500	8.15
	Pillow	50 × 50	453	3300	7.28
		50 × 50	455	3300	7.25

**Table 3:** TGA tests on Grafysorber^®^ samples from Nigeria.

Test	Initial Weight (mg)	Weight Loss (mg)	Adsorbent Weight (mg)	Adsorption (mg/mg)
Boom_1_1	10.243	−9.806	0.437	22.43
Boom_1_2	25.575	−24.264	1.311	18.51
Boom_1_3	35.045	−32.312	2.733	11.82
Boom_1_4	6.842	−6.426	0.415	15.45
Boom_1_5	31.848	−30.097	1.750	17.19
Boom_1_6	15.959	−14.982	0.977	15.34

**Table 4:** TGA analysis on pure PP samples.

Test	Initial Weight (mg)	Weight Loss (mg)	Heat of Fusion (mJ)	TGA Final T (°C)	Δ Weight (mg)
PP 1	4.519	−4.499	−280.9	550	0.020
PP 2	12.825	−6.773	−1146.0	400	6.052
PP 3	7.151	−7.144	−672.7	550	0.007
PP 4	8.418	−8.388	−720.9	550	0.030
PP 5	13.911	−13.895	−1271.4	550	0.016
PP 6	3.491	−3.501	−315.3	550	−0.010

**Table 5:** TGA tests on Grafysorber^®^ samples from Nigeria.

Test	Initial Weight (mg)	Weight Loss (mg)	Heat of Fusion (mJ)	Adsorbent Weight * (mg)	Adsorption (mg/mg)
Boom_5_1	12.841	−12.713	−167.22	2.548	4.99
Boom_5_2	20.966	−20.797	−236.01	3.256	6.39
Boom_5_3	19.938	−19.735	−282.23	3.732	5.29
Boom_5_4	20.751	−20.499	−327.26	4.195	4.89
Boom_5_5	19.938	−19.735	−282.23	3.732	5.29
Boom_5_6	11.659	−7.345	−101.26	1.870	3.93

* Estimated with calibration curve.
